# Autoencoder-like Sparse Non-Negative Matrix Factorization with Structure Relationship Preservation

**DOI:** 10.3390/e27080875

**Published:** 2025-08-19

**Authors:** Ling Zhong, Haiyan Gao

**Affiliations:** 1 School of Statistics and Data Science, Lanzhou University of Finance and Economics, Lanzhou 730020, China; zhongling@lzufe.edu.cn; 2 Key Laboratory of Digital Economy and Social Computing Science of Gansu, Lanzhou 730020, China

**Keywords:** structure relationship preservation, autoencoder-like, sparse constraint, non-negative matrix factorization, clustering

## Abstract

Clustering algorithms based on non-negative matrix factorization (NMF) have garnered significant attention in data mining due to their strong interpretability and computational simplicity. However, traditional NMF often struggles to effectively capture and preserve topological structure information between data during low-dimensional representation. Therefore, this paper proposes an autoencoder-like sparse non-negative matrix factorization with structure relationship preservation (ASNMF-SRP). Firstly, drawing on the principle of autoencoders, a “decoder-encoder” co-optimization matrix factorization framework is constructed to enhance the factorization stability and representation capability of the coefficient matrix. Then, a preference-adjusted random walk strategy is introduced to capture higher-order neighborhood relationships between samples, encoding multi-order topological structure information of the data through an optimal graph regularization term. Simultaneously, to mitigate the impact of noise and outliers, the $l_{2,1}$-norm is used to constrain the feature correlation between low-dimensional representations and the original data, preserving feature relationships between data, and a sparse constraint is imposed on the coefficient matrix via the inner product. Finally, clustering experiments conducted on 8 public datasets demonstrate that ASNMF-SRP consistently exhibits favorable clustering performance.

## 1. Introduction

Clustering aims to reveal intrinsic relationships between data through similarity measurement, and it is widely applied in various fields such as marketing [1], gene expression [2], and pattern recognition [3]. However, with the continuous increase in data scale, traditional clustering algorithms face challenges posed by the “curse of dimensionality”, including increased computational complexity, feature redundancy, and amplified noise. Consequently, researchers have proposed a collaborative optimization paradigm of “dimensionality reduction followed by clustering”. Matrix factorization, as a crucial dimensionality reduction method, factorizes the original data matrix into low-rank submatrices to achieve the goal of “parts representing the whole”. Common matrix factorization methods include Principal Component Analysis (PCA) [4], Singular Value Decomposition (SVD) [5], and non-negative matrix factorization (NMF) [6]. Due to advantages such as non-negativity constraints, computational simplicity, and fast implementation, NMF is frequently used in clustering analysis.

In recent years, to further enhance the performance of NMF, researchers have proposed many improved NMF algorithms. For example, Hoyer et al. [7] proposed sparse non-negative matrix factorization (SNMF), utilizing $l_{1}$-norm to improve the sparsity of NMF. Kong et al. [8] proposed robust non-negative matrix factorization (RNMF), replacing the Frobenius norm with the $l_{2,1}$-norm to enhance the algorithm’s robustness. Ding et al. [9] proposed orthogonal non-negative matrix factorization (ONMF), reducing interference from redundant information by imposing orthogonality constraints on the factorized submatrices. These NMF algorithms primarily focus on the reconstruction error after matrix factorization but overlook the spatial structural relationships within the original data. Studies have shown that introducing graph regularization techniques into NMF can not only optimize reconstruction error but also further capture local geometric structures between data [10]. Cai et al. [11] proposed the graph regularized non-negative matrix factorization algorithm (GNMF), encoding topological information between data into the objective function to effectively improve clustering performance. Wu et al. [12] proposed robust manifold non-negative matrix factorization (MNMEL21), which considers manifold learning of data and employs the *l*_2,1_-norm to mitigate interference from noise and outliers. Li et al. [13] proposed graph regularized non-negative low-rank matrix factorization (GNLMF), enhancing clustering accuracy on image data by capturing low-rank structures. Liu et al. [14] proposed graph regularized discriminative non-negative matrix factorization (GDNMF), which incorporates local geometric structure information between samples and data label information to effectively improve clustering performance on image data. To enhance the quality of graph learning, Huang et al. [15] proposed non-negative matrix factorization with adaptive graph learning (NMFAN), dynamically adjusting graph structure learning to effectively improve algorithm performance. Ren et al. [16] proposed semi-supervised symmetric non-negative matrix factorization with graph quality improvement (S^3^NMFGC), improving clustering performance by dynamically generating and adaptively updating graph learning results. Mohammadi et al. [17] proposed a semi-supervised multi-view clustering method based on adaptive symmetric NMF (SSA-SNMF), which improves the performance of the algorithm by introducing a multi-constraint optimization strategy.

In the process of graph learning, the quality of the learned graph structure directly affects the accuracy and robustness of clustering results. Currently, most graph learning methods are based on measuring Euclidean distances between samples. As the complexity of data internal structures increases, some samples may exhibit distant geometric distances, yet their local neighborhood topologies might show highly similar characteristics. Therefore, with the development of Graph Convolutional Neural Networks (GCNNs) [18], higher-order topological relationships between samples have gained widespread attention among researchers. Wang et al. [19] proposed a robust high-order graph learning algorithm for multi-view clustering (RHGL), which improves clustering accuracy by learning high-order graphs. Zhan et al. [20] proposed a multi-view clustering method with optimal high-order graph embedding (Co-MSE), which enhances the quality of graph learning by modeling high-order correlations. Additionally, Wang et al. [21] indicated that autoencoder-based methods can not only reduce data dimensionality but also further learn latent information between data points. Thus, from the perspective of “decoder-encoder” collaborative optimization, although the aforementioned improved NMF-based algorithms enhance clustering performance through different regularization terms, their objective functions only include the “decoder” component. They lack an explicit “encoder” structure with constraints on the coefficient matrix, thus preventing the coefficient matrix from fully uncovering latent information in the raw data.

Research indicates that introducing sparsity constraints into NMF can effectively enhance the clustering performance of algorithms [7,22]. Meng et al. [23] proposed sparse and orthogonally constrained dual-graph regularized non-negative matrix factorization (SODNMF), which incorporates dual-graph regularization, sparsity constraints, and orthogonality constraints into the objective function, significantly improving the algorithm’s performance. Peng et al. [24] proposed logarithmic sparse non-negative matrix factorization (LS-NMF), designing a $l_{\log}$-(pseudo) norm to achieve sparse constraints. Xiong et al. [25] proposed dual-graph regularized sparse robust and adaptive non-negative matrix factorization (DRGSNMF), adopting the $L_{2,p}$-norm to construct a sparse regularization term. Although the implementations of these algorithms differ, they all further enhance algorithm performance through sparse constraints.

Features often exhibit complex correlations or complementarity, such as in consumer behavior data. Therefore, in NMF, optimizing only the coefficient matrix may fail to effectively preserve structural relationships between features. To address this, Shang et al. [26] proposed dual-graph regularized non-negative matrix factorization (DNMF), which enhances the algorithm’s generalization capability by incorporating learning of the feature graph. Gu et al. [27] proposed a dual-graph regularized co-optimization clustering algorithm (DRCC), simultaneously considering the learning of both sample and feature graphs. Furthermore, to ensure the effective preservation of feature relationships from the original data matrix during dimensionality reduction, Hedjam et al. [28] proposed feature relationship-preserving non-negative matrix factorization (FR-NMF), and Salahian et al. [29] proposed a deep autoencoder non-negative matrix factorization with contrastive regularization and feature relationship preservation (DANMF-CRFR).

Therefore, this paper proposes an autoencoder-like sparse non-negative matrix factorization with structure relationship preservation (ASNMF-SRP). This method incorporates autoencoder principles into NMF, extending the constraints on the coefficient matrix from implicit to explicit. Through these dual constraints, it not only enhances the decomposition stability of the coefficient matrix but also further uncovers latent information in the raw data. Simultaneously, it preserves topological information in the original data space using higher-order graph regularization terms for both samples and features and incorporates an optimization term for feature relationship preservation. Finally, sparse optimization is applied to the coefficient matrix. Thus, ASNMF-SRP integrates autoencoder-like NMF, higher-order graph regularization, feature relationship preservation, and sparse constraints into a unified optimization framework.

The main contributions of the ASNMF-SRP algorithm are as follows:

(1) Optimization of the coefficient matrix. Firstly, this paper introduces an autoencoder-like NMF, adding an explicit “encoder” structure to constrain the coefficient matrix. Then, in the local structure learning process, a higher-order graph regularization method is proposed, enabling the algorithm to progressively extend from first-order to higher-order graph learning. Finally, an inner-product-based sparse constraint is introduced, preserving high-order collaborative relationships between data at the element level.

(2) Optimization of the basis matrix. This paper proposes a robust feature relationship preservation method, thereby establishing a topology-preserving relationship between the low-dimensional embedding space and the original high-dimensional data feature structure.

(3) This paper demonstrates the feasibility of ASNMF-SRP through convergence analysis, time complexity analysis, and other aspects. Simultaneously, in the experimental section, comparative analysis of clustering results, visualization analysis of low-dimensional representation data, parameter sensitivity analysis, and other experiments are conducted. Ultimately, the experimental results show that ASNMF-SRP exhibits excellent clustering performance.

The remaining work in this paper is organized as follows: Section 2 introduces some foundational work related to ASNMF-SRP. Section 3 primarily describes the derivation of the ASNMF-SRP algorithm, including the convergence and time complexity of submatrix update iterations. Section 4 presents a series of experiments conducted on eight public datasets. Section 5 provides a summary of the ASNMF-SRP algorithm.

## 2. Related Work

### 2.1. Non-Negative Matrix Factorization (NMF)

The objective of NMF is to factorize a given non-negative data matrix $X=\left(x_{1},x_{2},\cdots ,x_{n}\right)\in {\mathbb{R}}_{+}^{m\times n}$ into the product of two non-negative and low-rank matrices: a basis matrix $U\in {\mathbb{R}}_{+}^{m\times r}$ and a coefficient matrix $V\in {\mathbb{R}}_{+}^{r\times n}$, i.e., X≈UV, where $r\ll \min\left\{m,n\right\}$. During the factorization process, NMF optimizes the approximate representation by minimizing the reconstruction error. Therefore, it employs the Frobenius norm to define the objective function as follows:

(1) $$\min{\left\|X-UV\right\|}_{F}^{2},\;\;\;\;s.t.\;U\geq 0,\;V\geq 0$$

Here, ${\left\|\cdot \right\|}_{F}$ denotes the Frobenius norm of a matrix, which measures the reconstruction error after matrix factorization. For the optimization problem in Equation (1), Lee et al. [30] proposed an iterative algorithm based on multiplicative update rules, with the update formulas given by the following:

(2) $$U_{ij}\leftarrow U_{ij}\frac{{\left({XV}^{T}\right)}_{ij}}{{\left({UVV}^{T}\right)}_{ij}},\;\;\;V_{ij}\leftarrow V_{ij}\frac{{\left(U^{T}X\right)}_{ij}}{{\left(U^{T}UV\right)}_{ij}}$$

### 2.2. Robust Non-Negative Matrix Factorization (RNMF)

NMF measures reconstruction error based on Euclidean distance, making it highly susceptible to noise and outliers. To further enhance the robustness of the matrix factorization process, Kong et al. [8] proposed robust non-negative matrix factorization (RNMF), which employs the $l_{2,1}$-norm to assess the quality of data reconstruction. The objective function of RNMF is as follows:

(3) $$\min{\left\|X-UV\right\|}_{2,1},\;\;\;s.t.\;U\geq 0,\;V\geq 0$$

The update formulas for the basis matrix U and coefficient matrix V in Equation (3) are as follows:

(4) $$U_{ij}\leftarrow U_{ij}\frac{{\left(XG_{1}V^{T}\right)}_{ij}}{{\left(UVG_{1}V^{T}\right)}_{ij}},\;\;\;V_{ij}\leftarrow V_{ij}\frac{{\left(U^{T}XG_{1}\right)}_{ij}}{{\left(U^{T}UVG_{1}\right)}_{ij}}$$

Here, ***G***_1_ is a diagonal matrix with diagonal elements given by the following:

(5) $${\left(G_{1}\right)}_{jj}=1/{\left\|{\left(X-UV\right)}_{i}\right\|}_{2}$$

### 2.3. Graph Regularized Non-Negative Matrix Factorization (GNMF)

The standard NMF achieves the goal of “representing the whole by parts” by minimizing the reconstruction error, but this approach overlooks the latent structural information in the data. Therefore, Cai et al. [11] proposed graph regularized non-negative matrix factorization (GNMF). GNMF improves upon NMF by encoding the local geometric structure among samples into the objective function for optimization, thereby enhancing the representation quality of samples in the low-dimensional space. The objective function of GNMF is as follows:

(6) $$\min{\left\|X-UV\right\|}_{F}^{2}+\lambda \mathrm{Tr}\left({VLV}^{T}\right),\;\;\;s.t.\;U\geq 0,\;V\geq 0$$

Here, λ is a non-negative regularization parameter that adjusts the strength of graph learning. L is the Laplacian matrix, which satisfies L=D−W. In this context, W is the similarity matrix reflecting the relationships between samples, and the degree matrix $D=\mathrm{diag}(D_{11},D_{22},\cdots ,D_{nn})$ is a diagonal matrix formed by the row sums of W, i.e., $D_{ii}=\sum_{j}W_{ij}$. The update rules for GNMF are as follows:

(7) $$U_{ij}\leftarrow U_{ij}\frac{{\left({XV}^{T}\right)}_{ij}}{{\left({UVV}^{T}\right)}_{ij}},\;\;\;V_{ij}\leftarrow V_{ij}\frac{{\left(U^{T}X+\lambda WV\right)}_{ij}}{{\left(U^{T}UV+\lambda DV\right)}_{ij}}$$

## 3. Methodology

### 3.1. Autoencoder-like Non-Negative Matrix Factorization

In clustering analysis, the coefficient matrix V of NMF serves as the clustering indicator matrix. Therefore, the coefficient matrix V needs to effectively encode the structural features of the original data to ensure the accuracy of subsequent clustering. Inspired by the non-negative symmetric encoder-decoder method proposed by Sun et al. [31] in community detection and the principle of autoencoders, this paper imposes an explicit constraint on the coefficient matrix V, i.e., $V\approx f\left(X\right)=U^{T}X$. This approach not only improves the factorization stability of the coefficient matrix but also further uncovers the latent representations of the original data. The specific implementation process is as follows:

**Linear Decoder:**

(8) $$g\left(v\right)=Uv\to X\approx g\left(V\right)=UV\to {\left\|X-UV\right\|}_{F}^{2}$$

**Linear Encoder:**

(9) $$f\left(x\right)=U^{T}x\to V\approx f\left(X\right)=U^{T}X\to {\left\|V-U^{T}X\right\|}_{F}^{2}$$

In the “decoder-encoder” architecture, the coefficient matrix V acts as the encoder matrix. From Equations (8) and (9), it can be seen that the “decoder” part corresponds to standard NMF, aiming to minimize the reconstruction error between data, i.e., maximizing the approximation X≈UV. The “encoder” part, on the other hand, transforms the data matrix X into a distributed representation using the basis matrix $U\in {\mathbb{R}}_{+}^{m\times r}$, thereby imposing an explicit constraint on the coefficient matrix, i.e., $V\approx U^{T}X$.

By combining Equations (8) and (9), an autoencoder-like non-negative matrix factorization framework is obtained, with the following objective function:

(10) $$\min\underset{decoder}{\underset{︸}{{\left||X-UV|\right|}_{F}^{2}}}+\underset{encoder}{\underset{︸}{{\left||V-U^{T}X|\right|}_{F}^{2}}},\;\;\;s.t.\;U\geq 0,\;V\geq 0$$

**Figure 1:**
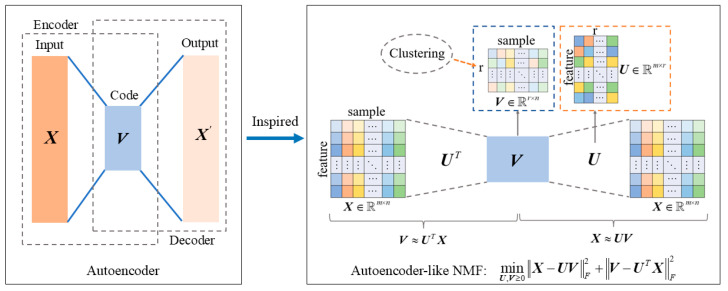
Schematic diagram of autoencoder-like non-negative matrix factorization.

Here, the decoder and encoder parts share the same basis matrix U, thereby forming a symmetry constraint. Figure 1 is a schematic diagram of Equation (10).

### 3.2. High-Order Graph Regularization

To further improve the quality of the data representation in the low-dimensional space, this paper introduces graph regularization techniques based on Equation (10) to learn the geometric structural information of the original high-dimensional space. Therefore, this paper captures the neighborhood relationships between samples by constructing a nearest neighbor graph. The first-order similarity matrix $W_{1}$ is defined as follows:

(11) $${\left(W_{1}\right)}_{ij}=\left\{\begin{array}{c} 1,\;x_{i}\in N_{k}\left(x_{j}\right)\;\mathrm{or}\;x_{j}\in N_{k}\left(x_{i}\right)\\ 0,\;\;\;\;otherwise \end{array}\right.$$

Here, $N_{k}\left(x_{i}\right)$ denotes the set of k-nearest data points to sample $x_{i}$. $x_{i}$ and $x_{j}$ are the i-th and j-th column samples of the original data matrix $X=\left(x_{1},x_{2},\cdots ,x_{n}\right)\in {\mathbb{R}}_{+}^{m\times n}$, respectively.

The coefficient matrix $V=\left(v_{1},v_{2},\cdots ,v_{n}\right)\in {\mathbb{R}}_{+}^{r\times n}$ is the low-dimensional representation to be optimized. According to manifold learning and spectral graph theory, the smoothness of this low-dimensional representation is given by the following:

(12) $$\begin{array}{l} Z=\frac{1}{2}\sum_{i,j=1}^{n}{\left\|v_{i}-v_{j}\right\|}^{2}{\left(W_{1}\right)}_{ij}\\ \;\;\;=\sum_{i=1}^{n}v_{i}v_{i}^{T}{\left(D_{1}\right)}_{ii}-\sum_{i,j=1}^{n}v_{i}v_{j}^{T}{\left(W_{1}\right)}_{ij}\\ \;\;\;=\mathrm{Tr}\left(VD_{1}V^{T}\right)-\mathrm{Tr}\left(VW_{1}V^{T}\right)\\ \;\;\;=\mathrm{Tr}\left(VL_{1}V^{T}\right) \end{array}$$

Here, $D_{1}$ is a diagonal matrix with ${\left(D_{1}\right)}_{ii}=\sum_{j}W_{ij}$. $L_{1}$ is defined as the first-order Laplacian matrix, which satisfies the equation $L_{1}=D_{1}-W_{1}$.

**Figure 2:**
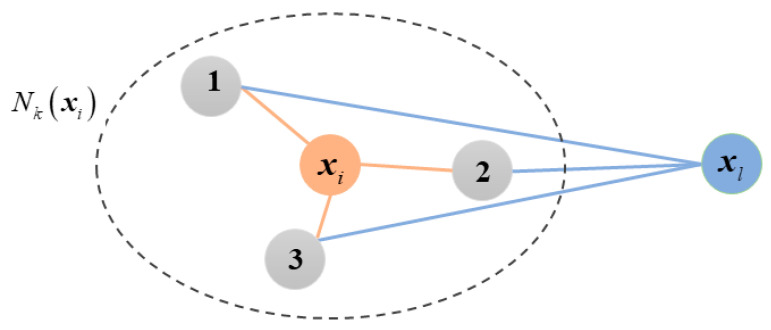
Example of associations between samples.

In real-world scenarios, samples often exhibit complex structural relationships. For instance, in online shopping, consumers from different regions may have no direct connection. However, by analyzing behavioral data, it is possible to identify neighborhood relationships where consumers share highly similar purchasing patterns. Such neighborhood relationships provide a scientific basis for businesses to segment target customer groups and implement precision marketing strategies. As shown in Figure 2, samples $x_{i}$ and $x_{l}$ are both connected to samples 1, 2, and 3, indicating that $x_{i}$ and $x_{l}$ share the same neighborhood structure. However, $x_{i}$ and $x_{l}$ are far apart and not within each other’s neighborhood sets. From Equation (11), it follows that $W_{il}=0$. Therefore, the first-order similarity matrix can only capture the neighborhood relationships between samples but fails to consider the similarity of their neighborhood structures. To further capture higher-order similarities between different neighborhood systems, this paper introduces a preference-adjusted random walk strategy. The random walk strategy [32,33] constructs sequences of sample points and encodes multi-step reachability into a higher-order similarity matrix, thereby reflecting higher-order structural information among samples. During the random walk process, the transition probability matrix $P_{ij}$ represents the probability of transitioning from sample $x_{i}$ to sample $x_{j}$, defined as follows:

(13) $$P_{ij}=\frac{a_{ij}}{k_{i}}$$

Here, $a_{ij}$ indicates the connection state between samples $x_{i}$ and $x_{j}$: if $x_{i}$ and $x_{j}$ are connected, then $a_{ij}=1$; otherwise, $a_{ij}=0$. $k_{i}$ represents the degree of sample $x_{i}$. Since $a_{ij}$ takes values of 0 or 1, $k_{i}$ here denotes the number of samples connected to $x_{i}$.

The transition probability matrix in Equation (13) is based on an equal-probability assumption, which often fails to accurately reflect the topological heterogeneity among samples when processing high-dimensional complex data. Therefore, this paper incorporates the Preferential Attachment (PA) model into Equation (13). By assigning connection preference weights between sample points, the transition probability matrix is further optimized to more realistically reflect higher-order neighborhood relationships among samples. Thus, the new expression is as follows:

(14) $$P_{ij}=\frac{PA_{simScore}}{Max\left(PA_{simScore}\right)}\times \frac{a_{ij}}{k_{i}}$$

Therefore, the d-th order similarity between samples $x_{i}$ and $x_{j}$ is as follows:

(15) $$S_{ij}^{d}=P_{ik}\times P_{kl}\times \cdots \times P_{zj}$$

where d=1,2,⋯,n. In practical applications, the value of d should not be too large, as an excessively large d may introduce noise interference, thereby affecting the quality of graph learning. The average of the d-th order adjacency matrices is as follows:

(16) $$W_{2}=\left(S^{1}+S^{2}+\cdots +S^{d}\right)/d$$

In Equation (16), $W_{2}$ is referred to as the second-order similarity matrix, which aims to capture higher-order neighborhood relationships between samples. The second-order Laplacian matrix is defined as $L_{2}=D_{2}-W_{2}$, where $D_{2}$ is a diagonal matrix with entries ${\left(D_{2}\right)}_{ii}=\sum_{j}W_{ij}$.

By considering both the first-order and second-order Laplacian matrices, the optimal Laplacian matrix is now defined as follows:

(17) $$L=\omega_{1}L_{1}+\omega_{2}L_{2}$$

where $w_{1}$ and $w_{2}$ are balancing parameters. Consequently, the optimal graph regularization term is given by the following:

(18) $$\underset{V\geq 0}{\min}\mathrm{Tr}\left(VLV^{T}\right)$$

### 3.3. Feature Relationship Preservation

**Figure 3:**
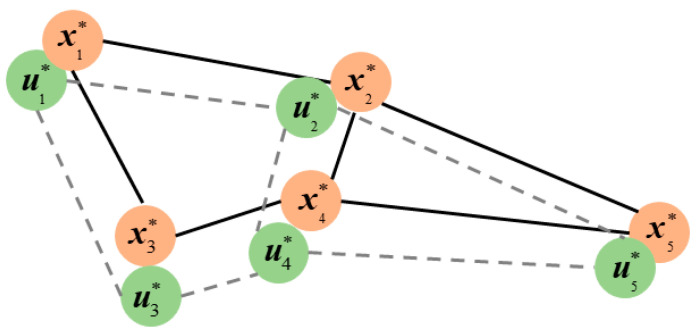
Example of feature relationship preservation in high-dimensional and low-dimensional spaces.

As data structures increase in complexity, feature information also plays a crucial role in clustering analysis. For example, when enterprises identify target customer groups, consumer characteristics, such as purchase frequency ($x_{1}^{*}$), interests ($x_{2}^{*}$), spending power ($x_{3}^{*}$), shopping preferences ($x_{4}^{*}$), and brand loyalty ($x_{5}^{*}$), are key dimensions. Therefore, when high-dimensional data are transformed into low-dimensional representations, preserving the intrinsic relationships between features becomes essential for effectively uncovering the latent structure of customer groups. This is illustrated in Figure 3.

In Figure 3, $x_{i}^{*}$ represents the *i*-th row vector of matrix X, corresponding to the *i*-th feature information in the original high-dimensional data, where i=1,2,3,4,5. $u_{i}^{*}$ represents the i-th row vector of the basis matrix U. As shown in Figure 3, the feature relationship between $x_{i}^{*}$ and $u_{i}^{*}$ remains consistent, which provides a theoretical basis for enterprises to identify target customer groups. Therefore, incorporating the feature relationships between data into the objective function for optimization can further preserve the feature relationships among the data.

${\left(XX^{T}\right)}_{ij}=\sum_{l=1}^{n}x_{il}x_{jl}$ represents the inner product between the i-th and j-th features of X. ${\left(UU^{T}\right)}_{ij}=\sum_{l=1}^{n}u_{il}u_{jl}$ represents the inner product between the i-th and j-th rows of the basis matrix U. To maintain consistency between feature relationships in the low-dimensional space and original data, we construct the feature preservation constraint term:

(19) $$\underset{U\geq 0}{\min}{\left\|XX^{T}-\beta \;UU^{T}\right\|}_{F}^{2}$$

where β is a non-negative balance parameter.

Equation (19) defines the reconstruction error based on the Frobenius norm. Therefore, when seeking to minimize the objective function value, Equation (19) is susceptible to interference from noise and outliers. To address this, this paper employs the $l_{2,1}$-norm to further optimize Equation (19), thereby enhancing its robustness. The specific expression is as follows:

(20) $$\underset{U\geq 0}{\min}{\left\|XX^{T}-\beta UU^{T}\right\|}_{2,1}$$

### 3.4. Sparsity of Coefficient Matrix

In the clustering process, the coefficient matrix V plays the role of a cluster indicator matrix. Therefore, the state of the coefficient matrix V affects the accuracy of clustering results. Imposing sparse constraints on the coefficient matrix V can make the representation of each sample in the low-dimensional space significantly correlated with only a few basis vectors, thereby weakening the interference of irrelevant information on clustering results. Common sparsity methods include: $l_{0}$-norm, $l_{1/2}$-norm, $l_{1}$-norm, $l_{2,1}$-norm, $l_{\log}$-(pseudo) norm, etc. A detailed introduction is shown in Table 1, where $v_{ij}$ represents the element in the i-th row and j-th column of the coefficient matrix $V\in {\mathbb{R}}_{+}^{r\times n}$.

The five common sparsity methods in Table 1 primarily impose element-wise regularization on the coefficient matrix V but overlook the correlations within the internal structure of V. This element-level separate optimization struggles to capture potential high-order collaborative relationships in the sample space. Therefore, this paper proposes a sparse constraint based on inner product penalty.

For the coefficient matrix $V=\left(v_{1},v_{2},\cdots ,v_{n}\right)\in {\mathbb{R}}_{+}^{r\times n}$, we have

(21) $$V^{T}V=\left[\begin{array}{cccc} \langle v_{1},v_{1}\rangle & \langle v_{1},v_{2}\rangle & \cdots & \langle v_{1},v_{n}\rangle \\ \langle v_{2},v_{1}\rangle & \langle v_{2},v_{2}\rangle & \cdots & \langle v_{2},v_{n}\rangle \\ \vdots & \vdots & \ddots & \vdots \\ \langle v_{n},v_{1}\rangle & \langle v_{n},v_{2}\rangle & \cdots & \langle v_{n},v_{n}\rangle \end{array}\right]$$

In Equation (21), the diagonal elements of $V^{T}V$ reflect autocorrelation, while the off-diagonal elements reflect correlations between different vectors. Here, to avoid interference from autocorrelation, we remove the diagonal elements based on Equation (21) and only retain the off-diagonal elements for sparsification. Thus, we can further obtain the following:

(22) $$\sum_{i=1}^{n}\sum_{j=1}^{n}\langle v_{i},v_{j}\rangle -\sum_{i=1}^{n}\langle v_{i},v_{i}\rangle =\sum_{i=1}^{n}\sum_{\begin{array}{l} j=1\\ j\neq i \end{array}}^{n}\langle v_{i},v_{j}\rangle =\mathrm{Tr}\left(V^{T}V1_{n}\right)-\mathrm{Tr}\left(V^{T}V\right)$$

where $1_{n}$ denotes an n×n matrix with all-one element.

Therefore, the sparse constraint term for the coefficient matrix V is as follows:

(23) $$\underset{V\geq 0}{\min}\left(\mathrm{Tr}\left(V^{T}V1_{n}\right)-\mathrm{Tr}\left(V^{T}V\right)\right)$$

### 3.5. Objective Function

**Figure 4:**
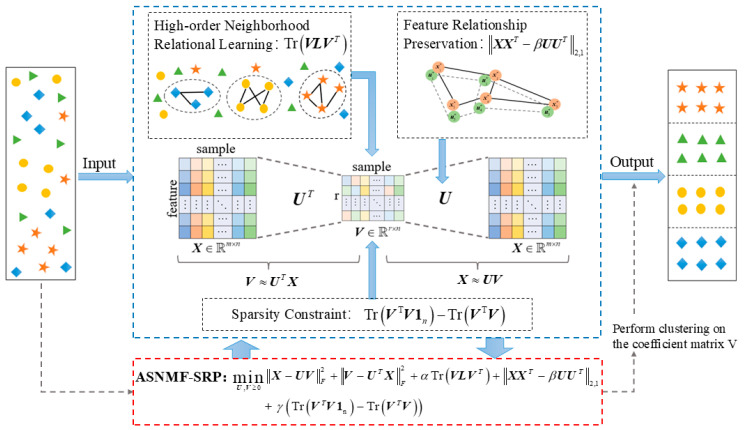
Schematic diagram of the ASNMF-SRP algorithm.

From Equations (10), (18), (20) and (23), the objective function of ASNMF-SRP is derived as follows:

(24) $$\begin{array}{l} \underset{U,V\geq 0}{\min}{\left\|X-UV\right\|}_{F}^{2}+{\left\|V-U^{T}X\right\|}_{F}^{2}+\alpha \mathrm{Tr}\left(VLV^{T}\right)+{\left\|{XX}^{T}-\beta {UU}^{T}\right\|}_{2,1}\\ \;\;\;\;\;+\lambda \left(\mathrm{Tr}\left(V^{T}V1_{n}\right)-\mathrm{Tr}\left(V^{T}V\right)\right) \end{array}$$

where α, β, and λ are all non-negative regularization parameters. Figure 4 is a schematic diagram of the ASNMF-SRP algorithm.

In the objective function Equation (24), each term plays an important role. The first term (${\left\|X-UV\right\|}_{F}^{2}$) aims to focus on the reconstruction error of matrix factorization. The second term (${\left\|V-U^{T}X\right\|}_{F}^{2}$) represents an explicit constraint on the coefficient matrix, thereby improving the factorization stability of the coefficient matrix. The third term ($\mathrm{Tr}\left({VLV}^{T}\right)$) encodes higher-order topological information between samples by constructing the optimal Laplacian matrix. The fourth term (${\left\|{XX}^{T}-\beta {UU}^{T}\right\|}_{2,1}$) ensures that the feature relationship structure of the original high-dimensional data can be preserved in the low-dimensional space after dimensionality reduction. The fifth term ($\mathrm{Tr}\left(V^{T}V1_{n}\right)-\mathrm{Tr}\left(V^{T}V\right)$) imposes a structured sparse constraint on the coefficient matrix by introducing a sparse regularization term in the form of inner product.

### 3.6. Optimization Algorithm

ASNMF-SRP employs multiplicative update rules to iteratively optimize the basis matrix U and coefficient matrix V. The augmented Lagrangian function of the objective function Equation (24) is as follows:

(25) $$\begin{array}{l} P\left(U,V\right)={\left\|X-UV\right\|}_{F}^{2}+{\left\|V-U^{T}X\right\|}_{F}^{2}+\alpha \mathrm{Tr}\left({VLV}^{T}\right)+{\left\|{XX}^{T}-\beta {UU}^{T}\right\|}_{2,1}\\ \;\;\;\;\;\;\;\;\;\;+\lambda \left(\mathrm{Tr}\left(V^{T}V1_{n}\right)-\mathrm{Tr}\left(V^{T}V\right)\right)-\mathrm{Tr}\left(\Lambda_{1}U^{T}\right)-\mathrm{Tr}\left(\Lambda_{2}V^{T}\right) \end{array}$$

where $\Lambda_{1}\in {\mathbb{R}}_{+}^{m\times r}$ and $\Lambda_{2}\in {\mathbb{R}}_{+}^{r\times n}$ denote the Lagrange multipliers for U and V, respectively.

Expanding Equation (25) and omitting terms independent of U and V, we obtain the following:

(26) $$\begin{array}{l} L\left(U,V\right)=\mathrm{Tr}\left(V^{T}U^{T}UV-2V^{T}U^{T}X\right)+\mathrm{Tr}\left(V^{T}V-2V^{T}U^{T}X+X^{T}UU^{T}X\right)\\ \;\;\;\;\;\;\;\;\;\;+\alpha \mathrm{Tr}\left(VLV^{T}\right)+\mathrm{Tr}\left(\beta^{2}UU^{T}G_{2}UU^{T}-2\beta UU^{T}G_{2}XX^{T}\right)\\ \;\;\;\;\;\;\;\;\;\;+\lambda \left(\mathrm{Tr}\left(V^{T}V1_{n}\right)-\mathrm{Tr}\left(V^{T}V\right)\right)-\mathrm{Tr}\left(\Lambda_{1}U^{T}\right)-\mathrm{Tr}\left(\Lambda_{2}V^{T}\right) \end{array}$$

where $G_{2}$ is a diagonal matrix whose diagonal elements are as follows:

(27) $${\left(G_{2}\right)}_{jj}=1/{\left\|{\left({XX}^{T}-\beta {UU}^{T}\right)}_{i}\right\|}_{2}$$

Taking partial derivatives of $L\left(U,V\right)$ with respect to U and V, respectively, gives the following:

(28) $$\begin{array}{l} \frac{\partial L}{\partial U}=2{UVV}^{T}-4{XV}^{T}+2{XX}^{T}U+2\beta^{2}{UU}^{T}G_{2}U+2\beta^{2}G_{2}{UU}^{T}U\\ \;\;\;\;\;-2\beta {XX}^{T}G_{2}U-2\beta G_{2}{XX}^{T}U-\Lambda_{1} \end{array}$$

(29) $$\frac{\partial L}{\partial V}=2U^{T}UV-4U^{T}X+2V+2\alpha VL+2\lambda V1_{n}-2\lambda V-\Lambda_{2}$$

To ensure the non-negativity of the coefficient matrix V, the optimal Laplacian matrix L in Equation (29) is factorized as $L=L^{+}-L^{-}$, where $L^{+}=(|L|+L)/2$ and $L^{-}=(|L|-L)/2$.

Equation (29) can be rewritten as follows:

(30) $$\frac{\partial L}{\partial V}=2U^{T}UV-4U^{T}X+2V+2\alpha VL^{+}-2\alpha VL^{-}+2\lambda V1_{n}-2\lambda V-\Lambda_{2}$$

According to the KKT conditions ${\left(\Lambda_{1}\right)}_{ij}U_{ij}=0$ and ${\left(\Lambda_{2}\right)}_{ij}V_{ij}=0$, we derive the following:

(31) $$\begin{array}{l} (2UVV^{T}-4XV^{T}+2XX^{T}U+2\beta^{2}UU^{T}G_{2}U+2\beta^{2}G_{2}UU^{T}U\\ \;\;\;\;\;\;\;\;\;\;\;\;\;\;\;\;\;\;\;\;-2\beta XX^{T}G_{2}U-2\beta G_{2}XX^{T}U-\Lambda_{1}{)}_{ij}U_{ij}=0 \end{array}$$

(32) $${\left(2U^{T}UV-4U^{T}X+2V+2\alpha VL^{+}-2\alpha VL^{-}+2\lambda V1_{n}-2\lambda V-\Lambda_{2}\right)}_{ij}V_{ij}=0$$

Therefore, we obtain the following:

(33) $$\begin{array}{l} (2UVV^{T}-4XV^{T}+2XX^{T}U+2\beta^{2}UU^{T}G_{2}U+2\beta^{2}G_{2}UU^{T}U\\ \;\;\;\;\;\;\;\;\;\;\;\;\;\;\;\;\;\;\;\;-2\beta XX^{T}G_{2}U-2\beta G_{2}XX^{T}U-\Lambda_{1}{)}_{ij}U_{ij}^{2}=0 \end{array}$$

(34) $${\left(2U^{T}UV-4U^{T}X+2V+2\alpha VL^{+}-2\alpha VL^{-}+2\lambda V1_{n}-2\lambda V-\Lambda_{2}\right)}_{ij}V_{ij}^{2}=0$$

From Equations (33) and (34), the update formulas for U and V are as follows:

(35) $$U_{ij}^{t+1}\leftarrow U_{ij}^{t}\sqrt{\frac{{\left(2{XV}^{T}+\beta {XX}^{T}G_{2}U+\beta G_{2}{XX}^{T}U\right)}_{ij}}{{\left({UVV}^{T}+{XX}^{T}U+\beta^{2}{UU}^{T}G_{2}U+\beta^{2}G_{2}{UU}^{T}U\right)}_{ij}}}$$

(36) $$V_{ij}^{t+1}\leftarrow V_{ij}^{t}\sqrt{\frac{{\left(2U^{T}X+\alpha VL^{-}+\lambda V\right)}_{ij}}{{\left(U^{T}UV+V+\alpha VL^{+}+\lambda V1_{n}\right)}_{ij}}}$$

### 3.7. Convergence Analysis

In this section, the convergence of U and V under the update rules in Equations (35) and (36) in the ASNMF-SRP algorithm is proven using the auxiliary function method.

Firstly, we prove that the coefficient matrix V is non-increasing under the update rule in Equation (36). According to the objective function Equation (24), we can derive a functional $F_{ij}\left(V_{ij}\right)$ regarding the coefficient matrix V as follows:

(37) $$F_{ij}(V_{ij})=\mathrm{Tr}(V^{T}U^{T}UV-4V^{T}U^{T}X+V^{T}V+\alpha VL^{+}V^{T}-\alpha VL^{-}V^{T}+\lambda V^{T}V1_{n}-\lambda V^{T}V)$$

**Proposition** **1.** *The constructed function* $G\left(V_{ij},V_{ij}^{t}\right)$ *is an auxiliary function of* $F_{ij}\left(V_{ij}\right)$.

(38) $$\begin{array}{l} G\left(V_{ij},V_{ij}^{t}\right)=\sum_{ij}{\left(U^{T}UV^{t}\right)}_{ij}\frac{V_{ij}^{2}}{V_{ij}^{t}}-4\sum_{ij}{\left(U^{T}X\right)}_{ij}V_{ij}^{t}\left(1+\log\frac{V_{ij}}{V_{ij}^{t}}\right)\\ \;\;\;\;\;\;\;\;\;+\sum_{ij}V_{ij}^{2}+\alpha \sum_{ij}{\left(V^{t}L^{+}\right)}_{ij}\frac{V_{ij}^{2}}{V_{ij}^{t}}-\alpha \sum_{ijl}\left(L_{jl}^{-}V_{ij}^{t}V_{il}^{t}\right)\left(1+\log\frac{V_{jl}V_{il}}{V_{jl}^{t}V_{il}^{t}}\right)\\ \;\;\;\;\;\;\;\;\;+\lambda \sum_{ij}{\left(V^{t}1_{n}\right)}_{ij}\frac{V_{ij}^{2}}{V_{ij}^{t}}-\lambda \sum_{ij}V_{ij}^{t}V_{ij}^{t}\left(1+\log\frac{V_{ij}V_{ij}}{V_{ij}^{t}V_{ij}^{t}}\right) \end{array}$$

**Proof.**  When $V_{ij}=V_{ij}^{t}$, we have $G\left(V_{ij},V_{ij}\right)=F_{ij}\left(V_{ij}\right)$. The following proves the case $G\left(V_{ij},V_{ij}^{t}\right)\geq F_{ij}\left(V_{ij}\right)$.

When A and B are symmetric matrices, we have the following:

(39) $$\begin{array}{l} \sum_{i=1}^{n}\sum_{j=1}^{m}\frac{{\left(AS^{t}B\right)}_{ij}S_{ij}^{2}}{S_{ij}^{t}}\geq \mathrm{Tr}\left(S^{T}ASB\right),\\ \forall \;A\in {\mathbb{R}}_{+}^{n\times n},\;B\in {\mathbb{R}}_{+}^{m\times m},\;S^{t}\in {\mathbb{R}}_{+}^{n\times m},\;S\in {\mathbb{R}}_{+}^{n\times m} \end{array}$$

From Equation (39), we obtain the following:

(40) $$\mathrm{Tr}\left(V^{T}U^{T}UV\right)\leq \sum_{ij}{\left(U^{T}UV^{t}\right)}_{ij}\frac{V_{ij}^{2}}{V_{ij}^{t}}$$

(41) $$\mathrm{Tr}\left(V^{T}V\right)\leq \sum_{ij}V_{ij}^{2}$$

(42) $$\alpha \mathrm{Tr}\left(VL^{+}V^{T}\right)\leq \alpha \sum_{ij}{\left(V^{t}L^{+}\right)}_{ij}\frac{V_{ij}^{2}}{V_{ij}^{t}}$$

(43) $$\lambda \mathrm{Tr}\left(V^{T}V1_{n}\right)\leq \lambda \sum_{ij}{\left(V^{t}1_{n}\right)}_{ij}\frac{V_{ij}^{2}}{V_{ij}^{t}}$$

From $x>1+\log x\left(x>0\right)$, we obtain the following:

(44) $$-4\mathrm{Tr}\left(V^{T}U^{T}X\right)\;=-4\mathrm{Tr}\left(X^{T}UV\right)\leq -4\sum_{ij}{\left(U^{T}X\right)}_{ij}V_{ij}^{t}\left(1+\log\frac{V_{ij}}{V_{ij}^{t}}\right)$$

(45) $$-\alpha \mathrm{Tr}\left(VL^{-}V^{T}\right)\leq -\alpha \sum_{ijl}\left(L_{jl}^{-}V_{ij}^{t}V_{il}^{t}\right)\left(1+\log\frac{V_{jl}V_{il}}{V_{jl}^{t}V_{il}^{t}}\right)$$

(46) $$-\lambda \mathrm{Tr}\left(V^{T}V\right)\leq -\lambda \sum_{ij}V_{ij}^{t}V_{ij}^{t}\left(1+\log\frac{V_{ij}V_{ij}}{V_{ij}^{t}V_{ij}^{t}}\right)$$

From Equations (40)–(46), it follows that $G\left(V_{ij},V_{ij}^{t}\right)\geq F_{ij}\left(V_{ij}\right)$. Therefore, $G\left(V_{ij},V_{ij}^{t}\right)$ is an auxiliary function of $F_{ij}\left(V_{ij}\right)$, and Proposition 1 is proven.

Let $\frac{\partial G\left(V,V^{t}\right)}{\partial V_{ij}}=0$, we obtain the following:

(47) $$V_{ij}^{t+1}\leftarrow V_{ij}^{t}\sqrt{\frac{{\left(2U^{T}X+\alpha VL^{-}+\lambda V\right)}_{ij}}{{\left(U^{T}UV+V+\alpha VL^{+}+\lambda V1_{n}\right)}_{ij}}}$$

Thus, the coefficient matrix V in the ASNMF-SRP algorithm is non-increasing under the update rule in Equation (36). Similarly, it can be proven that the basis matrix U is non-increasing under the update rule in Equation (35). Therefore, U and V converge under the update rules in Equations (35) and (36). The specific implementation of the ASNMF-SRP algorithm is described in Algorithm 1.
**Algorithm 1** ASNMF-SRP
**Input:** Initial matrix $X=\left(x_{1},x_{2},\cdots ,x_{n}\right)\in {\mathbb{R}}_{+}^{m\times n}$, number of classes r, neighborhood parameter k, regularization parameters α, β and λ, balance parameters $w_{1}$ and $w_{2}$ parameter d, threshold ε, maximum iterations t.
**Output:** Basis matrix U and coefficient matrix V.
1. Initialization: t=0, Randomly generate basis matrix $U\in {\mathbb{R}}^{m\times r}$ and coefficient matrix $V\in {\mathbb{R}}^{r\times n}$;
2. Obtain optimal Laplacian matrix L according to Equations (11)–(17);
3. For t=1,2,3,⋯,maxIter
4. $U_{ij}^{t+1}\leftarrow U_{ij}^{t}\sqrt{\frac{{\left(2{XV}^{T}+\beta {XX}^{T}G_{2}U+\beta G_{2}{XX}^{T}U\right)}_{ij}}{{\left({UVV}^{T}+{XX}^{T}U+\beta^{2}{UU}^{T}G_{2}U+\beta^{2}G_{2}{UU}^{T}U\right)}_{ij}}}$;
5. $V_{ij}^{t+1}\leftarrow V_{ij}^{t}\sqrt{\frac{{\left(2U^{T}X+\alpha VL^{-}+\lambda V\right)}_{ij}}{{\left(U^{T}UV+V+\alpha VL^{+}+\lambda V1_{n}\right)}_{ij}}}$;
6. if ${\left\|U^{t}-U^{t-1}\right\|}_{\infty }\leq \varepsilon$ and ${\left\|V^{t}-V^{t-1}\right\|}_{\infty }\leq \varepsilon$
Break and return (U,V);
7. End if
8. End for

□

### 3.8. Time Complexity Analysis

When inputting a data matrix $X\in {\mathbb{R}}^{m\times n}$, where m is the feature dimension of samples and n is the number of samples, assume the number of classes for this dataset is r, and $r\ll \min\left\{m,n\right\}$. After t iterations, the time complexity of ASNMF-SRP is as follows:

(1) The autoencoder-like NMF part: complexities for updating U and V are $O\left(tm^{2}r+tmr^{2}\right)$ and $O\left(tm^{2}r+tmr^{2}\right)$;

(2) The higher-order graph regularization part: complexity is $O\left(mn^{2}+me^{d}\right)$, where e denotes the average degree of nodes;

(3) The feature preservation part: complexity is $O\left(tm^{2}r+t\left(m^{2}+m\right)\right)$;

(4) The sparse constraint term: complexity is $O\left(tn^{2}r\right)$.

Therefore, the overall time complexity of ASNMF-SRP is $O\left(tm^{2}r+t\left(m^{2}+m\right)+tn^{2}r+mn^{2}+me^{d}\right)$.

## 4. Experiments

In this section, clustering experiments will be conducted on 8 public datasets to evaluate the clustering performance of the ASNMF-SRP algorithm. The experiments are implemented in Python 3.11 with the computer environment being Intel(R) Core (TM) i5-1135G7 @ 2.40 GHz 2.42 GHz, 16 GB RAM, and Windows 11 64-bit operating system.

### 4.1. Dataset

To verify the effectiveness of the algorithm, we selected 8 public datasets for comparative experiments. MSRA25 [37] dataset contains 1799 face images from 12 individuals. Semeion, Krvs, PenDigits, and Vehicle datasets are from the UCI Machine Learning Repository (https://archive.ics.uci.edu (accessed on 23 March 2025)). Hitech dataset is from the CLUTO toolkit (https://conservancy.umn.edu/items/4fbef165-f964-41ed-a239-86a8f931ffbe (accessed on 26 March 2025)). COIL20 (http://www.cs.columbia.edu/CAVE/software/softlib/coil-20.php (accessed on 15 December 2024)) and COIL100 (http://www.kaggle.com/jessicali9530/coil100/downloads/coil100.zip/2 (accessed on 15 December 2024)) datasets record images of 20 and 100 objects, respectively. The basic information of these 8 datasets is shown in Table 2.

### 4.2. Clustering Performance Evaluation Metrics

To compare the clustering performance of various algorithms, this paper selects four commonly used clustering performance evaluation metrics. Among them, larger values for these four clustering evaluation metrics indicate better clustering performance of the corresponding algorithm.

#### 4.2.1. Clustering Accuracy (ACC)

(48) $$\mathrm{ACC}=\frac{\sum_{i=1}^{n}\delta \left(map\left(s_{i}\right),r_{i}\right)}{n}$$

where n is the total number of samples in the input data, $r_{i}$ is the true class label of the data, $s_{i}$ represents the result after clustering by the algorithm, and $map\left(\cdot \right)$ denotes the mapping function. The expression $\delta \left(map\left(s_{i}\right),r_{i}\right)$ in Equation (48) is as follows:

(49) $$\delta \left(map\left(s_{i}\right),r_{i}\right)=\left\{\begin{array}{c} 1,\;map\left(s_{i}\right)=r_{i}\\ 0,\;map\left(s_{i}\right)\neq r_{i} \end{array}\right.$$

From Equations (48) and (49), ACC is an evaluation metric used to measure the consistency between clustering results and true labels, with a value range of $\left[0,1\right]$.

#### 4.2.2. Adjusted Rand Index (ARI)

(50) $$\mathrm{ARI}=\frac{\sum_{ij}\left(\begin{array}{c} n_{ij}\\ 2 \end{array}\right)-\left[\sum_{i}\left(\begin{array}{c} x_{i}\\ 2 \end{array}\right)\sum_{j}\left(\begin{array}{c} y_{i}\\ 2 \end{array}\right)\right]/\left(\begin{array}{c} n\\ 2 \end{array}\right)}{0.5\left[\sum_{i}\left(\begin{array}{c} x_{i}\\ 2 \end{array}\right)+\sum_{j}\left(\begin{array}{c} y_{i}\\ 2 \end{array}\right)\right]-\left[\sum_{i}\left(\begin{array}{c} x_{i}\\ 2 \end{array}\right)\sum_{j}\left(\begin{array}{c} y_{i}\\ 2 \end{array}\right)\right]/\left(\begin{array}{c} n\\ 2 \end{array}\right)}$$

where $x_{i}$ represents the total number of samples in the i-th cluster after clustering, $y_{i}$ represents the total number of samples in the true j-th class, and $n_{ij}$ denotes the total number of samples common to both the cluster $x_{i}$ and the true class $y_{j}$. From Equation (50), the value range of ARI is $\left[-1,1\right]$.

#### 4.2.3. Normalized Mutual Information (NMI)

(51) $$\mathrm{NMI}\left(N,N^{*}\right)=\frac{\mathrm{MI}\left(N,N^{*}\right)}{\max\left(H\left(N\right),H\left(N^{*}\right)\right)}$$

where $N^{*}$ represents the data labels obtained through clustering, N represents the actual data labels, and $H\left(\cdot \right)$ is defined as the entropy function.

The mutual information $\mathrm{MI}\left(\cdot \right)$ expression is as follows:

(52) $$\mathrm{MI}\left(N,N^{*}\right)=\sum_{n_{i}\in N,n_{j}^{*}\in N^{*}}p\left(n_{i},n_{j}^{*}\right)\log\frac{p\left(n_{i},n_{j}^{*}\right)}{p\left(n_{i}\right)p\left(n_{j}^{*}\right)}$$

where $p\left(n_{i}\right)$ and $p\left(n_{j}^{*}\right)$ represent the marginal probabilities of the true class and clustering results, respectively, and $p\left(n_{i},n_{j}^{*}\right)$ is the joint probability of the true class $n_{i}$ and cluster $n_{j}^{*}$. From Equations (51) and (52), the value range of NMI is $\left[0,1\right]$.

#### 4.2.4. Clustering Purity (PUR)

(53) $$\mathrm{PUR}=\sum_{i=1}^{r}\frac{\max_{j}\left(n_{i}^{j}\right)}{n}$$

where $n_{i}$ represents the total number of samples, r is the number of clusters, and $n_{i}^{j}$ represents the number of samples in cluster i that belong to the true class j. From Equation (53), the value range of PUR is $\left[0,1\right]$.

### 4.3. Comparison Algorithms and Parameter Settings

The detailed descriptions of the 9 algorithms participating in the comparative experiments are as follows:

(1) NMF [6] factorizes the original non-negative matrix into the product of two non-negative low-rank submatrices to achieve the goal of representing the whole by parts.

(2) ONMF [9] imposes orthogonality constraints on the factorized submatrices based on NMF.

(3) Hx-NMF [38] combines logarithm with NMF to improve the robustness of the algorithm.

(4) EMMF [39] is an algorithm based on entropy minimization matrix factorization.

(5) GNMF [11] encodes the local geometric structure between data into the objective function to improve the quality of low-dimensional representations.

(6) RMMMF [40] uses the $l_{2,1}$-norm to enhance the algorithm’s robustness.

(7) DRCC [27] jointly optimizes the local geometric structures of samples and features by incorporating them into the objective function.

(8) FR-NMF [28] improves the clustering performance of the algorithm by introducing a feature preservation term.

(9) LS-NMF [24] imposes sparse constraints on the basis matrix and coefficient matrix using the $l_{log}$-(pseudo) norm.

In the experimental process, we perform a grid search over the algorithm’s regularization parameter in the range $\left\{10^{-4},10^{-3},10^{-2},10^{-1},10^{0},10^{1},10^{2},10^{3}\right\}$.

During the experiments, the parameter values for the higher-order graph regularization parameter α, feature relationship preservation regularization parameter β, and sparse constraint regularization parameter λ in the ASNMF-SRP algorithm are shown in Table 3.

### 4.4. Results and Analysis

Due to fluctuations in the clustering results of various algorithms, to obtain representative clustering outcomes, each algorithm was executed 20 times on the 8 datasets during the experiments. The mean and standard deviation were calculated as the final comparative clustering results. Table 4, Table 5, Table 6 and Table 7 present the specific values of ACC, ARI, NMI, and PUR for these 10 algorithms on the 8 datasets, respectively. In the tables, bold numbers indicate the optimal clustering results on that dataset, and the I-P (Improvement Percentage) column shows the percentage by which the ASNMF-SRP algorithm improves over the best result among the other 9 algorithms.

By comparing the data in Table 4, Table 5, Table 6 and Table 7, it can be seen that ASNMF-SRP exhibits excellent clustering performance. The specific data analysis is as follows:

(1) On image datasets (MSRA25, Semeion, COIL20, and COIL100), the values of all four clustering evaluation metrics for ASNMF-SRP are higher than those of the other nine algorithms. This indicates that ASNMF-SRP holds certain advantages in handling clustering problems related to images.

(2) On non-image datasets (Krvs, Hitech, PenDigits, and Vehicle), ASNMF-SRP fails to achieve optimal values in ACC on the Hitech dataset and in ARI and NMI on the Vehicle dataset. However, in most cases, ASNMF-SRP demonstrates optimal performance.

(3) FR-NMF incorporates an additional feature relationship preservation term compared to NMF. According to the data in Table 4, Table 5, Table 6 and Table 7, FR-NMF outperforms NMF in clustering performance on some datasets. ASNMF-SRP not only includes feature relationship preservation but also incorporates components such as autoencoder-like NMF, higher-order graph regularization, and sparse constraints. The data in Table 4, Table 5, Table 6 and Table 7 show that ASNMF-SRP achieves higher values across all four clustering evaluation metrics on these eight datasets compared to NMF and FR-NMF. This demonstrates that through the synergistic effects of various regularization terms, ASNMF-SRP not only enhances clustering performance but also improves generalization capability.

This paper employs the t-SNE method to project the low-dimensional representation matrices obtained by the ten algorithms on the MSRA25, COIL20, and Hitech datasets into a two-dimensional space for visualization.

**Figure 5:**
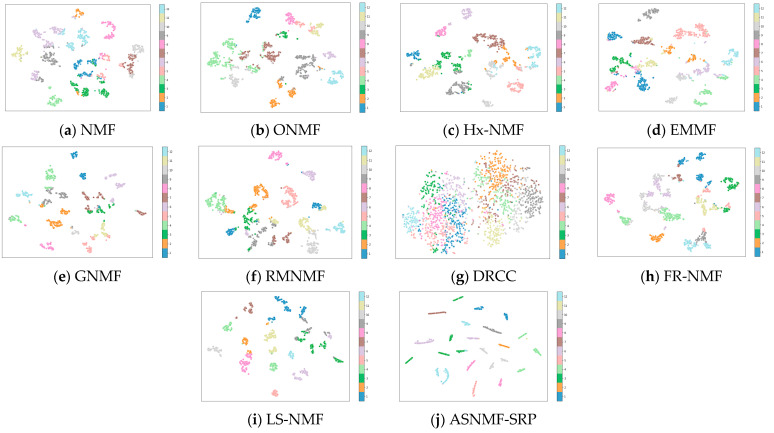
Visual comparison of low-dimensional representation matrices of various algorithms on the MSRA25 dataset.

**Figure 6:**
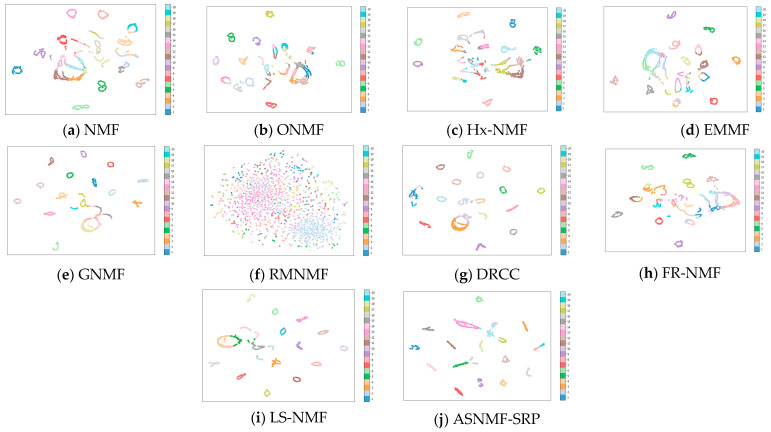
Visual comparison of low-dimensional representation matrices of various algorithms on the COIL20 dataset.

**Figure 7:**
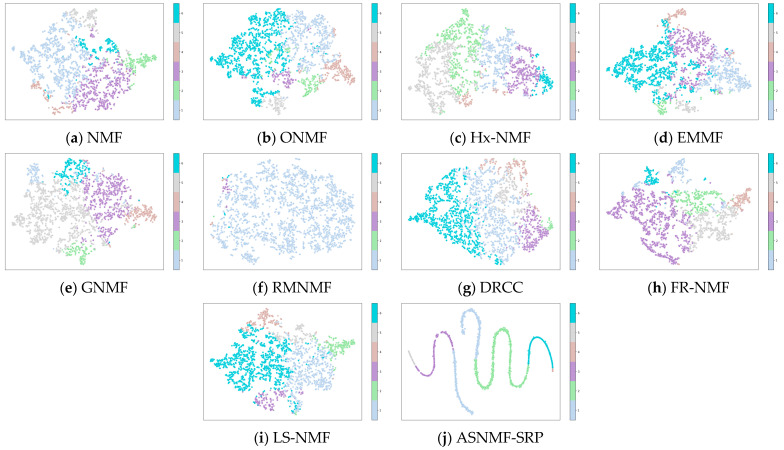
Visual comparison of low-dimensional representation matrices of various algorithms on the Hitech dataset.

From Figure 5, Figure 6 and Figure 7, it can be observed that on the MSRA25, COIL20, and Hitech datasets, the low-rank representation matrix obtained after dimensionality reduction using the ASNMF-SRP algorithm exhibits stronger discriminative power. Therefore, the ASNMF-SRP algorithm demonstrates excellent clustering performance.

### 4.5. Analysis of the Impact of Autoencoder-like NMF on Clustering Performance

Unlike traditional NMF, the objective function of ASNMF-SRP includes a “decoder- encoder” module. To investigate the influence of this autoencoder-like NMF architecture on clustering performance, we removed the “encoder” part in Equation (24), causing ASNMF-SRP to degenerate into ASNMF-SRP-1. The objective function of ASNMF-SRP-1 is as follows:

(54) $$\underset{U,V\geq 0}{\min}{\left\|X-UV\right\|}_{F}^{2}+\alpha \mathrm{Tr}\left({VLV}^{T}\right)+{\left\|{XX}^{T}-\beta {UU}^{T}\right\|}_{2,1}+\lambda \left(\mathrm{Tr}\left(V^{T}V1_{n}\right)-\mathrm{Tr}\left(V^{T}V\right)\right)$$

From Table 8, it can be seen that the clustering performance of ASNMF-SRP is significantly better than that of ASNMF-SRP-1. On the clustering evaluation metrics ACC, ARI, NMI, and PUR, it shows average improvements of 12.03%, 17.02%, 10.81%, and 10.56%, respectively. This indicates that introducing an NMF component with a structure similar to an autoencoder has a positive effect on ASNMF-SRP, effectively enhancing its clustering performance.

### 4.6. Analysis of the Impact of Higher-Order Graph Regularization on Clustering Performance

To further examine the influence of higher-order neighborhood relationships among samples on clustering performance, we set $w_{2}=0$ in Equation (17), thereby reducing ASNMF-SRP to ASNMF-SRP-2. At this point, the objective function of ASNMF-SRP-2 is as follows:

(55) $$\begin{array}{l} \underset{U,V\geq 0}{\min}{\left\|X-UV\right\|}_{F}^{2}+{\left\|V-U^{T}X\right\|}_{F}^{2}+\alpha \mathrm{Tr}\left(VL_{1}V^{T}\right)+{\left\|XX^{T}-\beta UU^{T}\right\|}_{2,1}\\ \;\;\;\;\;+\lambda \left(\mathrm{Tr}\left(V^{T}V1_{n}\right)-\mathrm{Tr}\left(V^{T}V\right)\right) \end{array}$$

From Table 9, it can be observed that ASNMF-SRP exhibits superior clustering performance over ASNMF-SRP-2 on most datasets, indicating its stronger generalization ability. This also suggests that higher-order neighborhood relationships among samples can influence the clustering performance of the algorithm. However, on a few datasets (e.g., Krvs), ASNMF-SRP does not perform well. Therefore, future work should further explore how to design an optimal Laplacian matrix to effectively encode multi-order topological structure information, thereby improving the algorithm’s universality.

### 4.7. Robustness Analysis of ASNMF-SRP

To evaluate the robustness of ASNMF-SRP, we added salt-and-pepper noise with intensities of 10%, 20%, 30%, and 40% to the MSRA25, COIL20, and COIL100 datasets. To further compare the contributions of Equations (19) and (20) to the robustness of ASNMF-SRP, we conducted comparative experiments between ASNMF-SRP and ASNMF-SRP-3. The objective function of ASNMF-SRP-3 is as follows:

(56) $$\begin{array}{l} \underset{U,V\geq 0}{\min}{\left\|X-UV\right\|}_{F}^{2}+{\left\|V-U^{T}X\right\|}_{F}^{2}+\alpha \mathrm{Tr}\left(VLV^{T}\right)+{\left\|XX^{T}-\beta UU^{T}\right\|}_{F}^{2}\\ \;\;\;\;\;+\lambda \left(\mathrm{Tr}\left(V^{T}V1_{n}\right)-\mathrm{Tr}\left(V^{T}V\right)\right) \end{array}$$

**Figure 8:**
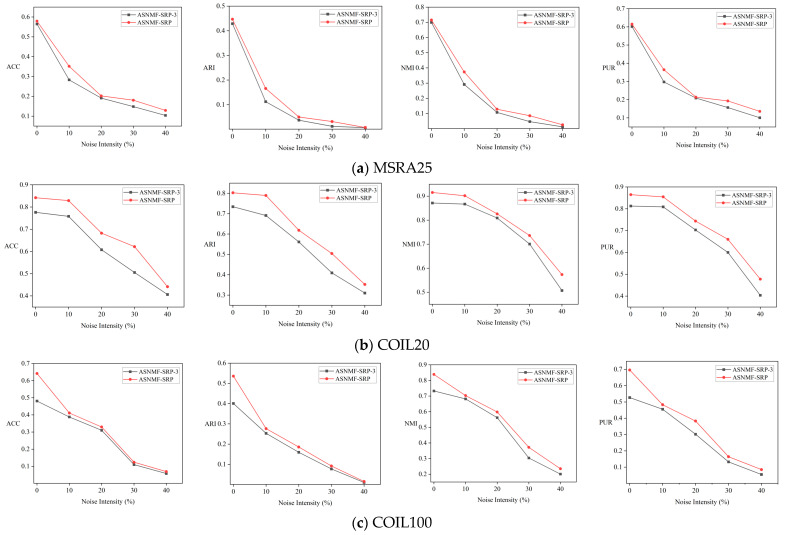
Comparison of clustering results between ASNMF-SRP and ASNMF-SRP-3 under different noise intensities.

From Figure 8, it can be seen that as the noise intensity increases, the clustering evaluation metric values of both ASNMF-SRP and ASNMF-SRP-3 show a declining trend. However, the clustering evaluation metric values of ASNMF-SRP are consistently higher than those of ASNMF-SRP-3. Therefore, adopting the $l_{2,1}$-norm to measure reconstruction errors in the “feature relationship preservation” component can enhance the robustness of ASNMF-SRP.

### 4.8. Parameter Sensitivity Analysis

**Figure 9:**
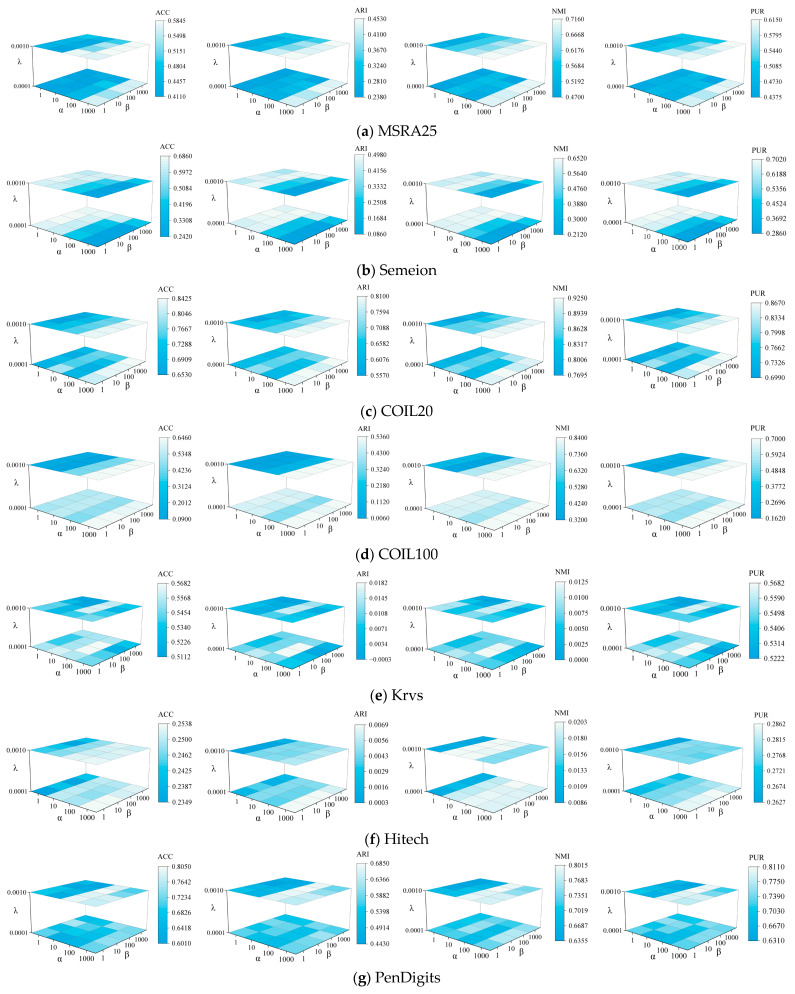

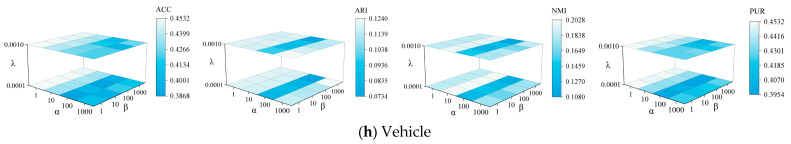
Parameter sensitivity analysis of ASNMF-SRP on various datasets.

ASNMF-SRP has three hyperparameters: the higher-order graph regularization coefficient α, the balance parameter for feature relationship preservation β, and the regularization parameter for sparse constraint λ. In the parameter sensitivity analysis experiment, the value ranges for α and β were set to $\left\{10^{0},10^{1},10^{2},10^{3}\right\}$, and the range for λ was $\left\{10^{-4},10^{-3}\right\}$. The parameter sensitivity concerning α, β, and λ is shown in Figure 9.

From Figure 9, we can obtain the following:

(1) On the MSRA25, COIL20, COIL100, and Hitech datasets, ASNMF-SRP demonstrates favorable clustering performance when the higher-order regularization parameter α=1000; on the Semeion and Vehicle datasets, ASNMF-SRP exhibits good clustering performance when parameter α takes smaller values.

(2) With parameters α and β determined, different values of the sparse constraint regularization parameter λ significantly affect the clustering performance of the ASNMF-SRP algorithm. For example, on the COIL100 dataset, when λ=0.0001, the clustering results of ASNMF-SRP overall outperform those when λ=0.001.

(3) α and λ jointly constrain the coefficient matrix V, where α preserves the manifold structure of samples while λ balances the strength of sparsity, both optimizing the coefficient matrix through synergistic effects. Simultaneously, α and β collaboratively optimize the local geometric structure of data in both sample space and feature space. In parameter settings, the values of α and β are generally greater than that of λ to enhance manifold structure preservation capability, while the value of λ is kept relatively small to ensure appropriate sparse constraint intensity.

### 4.9. Empirical Convergence

This section will experimentally analyze the convergence of the ASNMF-SRP algorithm on 8 datasets to further verify the accuracy of the theoretical analysis in Section 3.7. In the empirical convergence experiment, the values of hyperparameters α, β, and λ in ASNMF-SRP remain consistent with those in Table 3.

**Figure 10:**
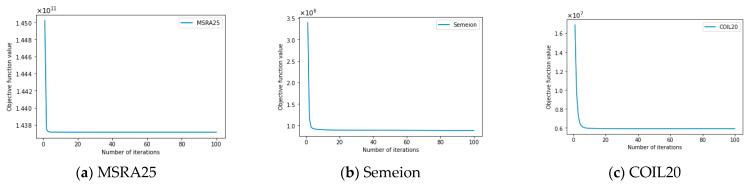

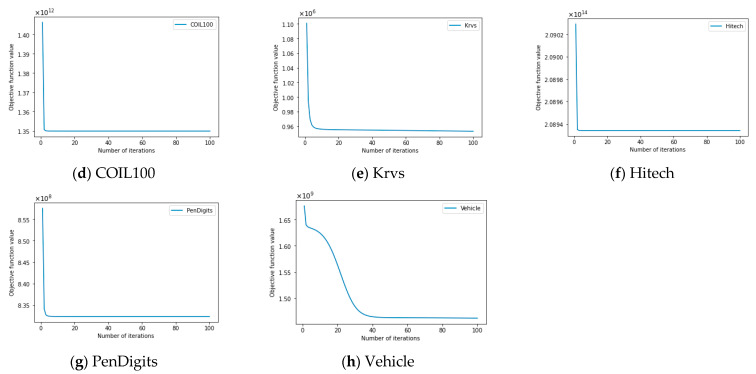
Convergence curves of ASNMF-SRP on various datasets.

From Figure 10, it can be observed that the ASNMF-SRP algorithm achieves convergence on all eight datasets. Specifically, on the MSRA25, Semeion, COIL20, COIL100, Krvs, Hitech, and PenDigits datasets, the ASNMF-SRP algorithm exhibits a relatively fast convergence rate during the initial iterations, with its corresponding objective function value rapidly decreasing, ultimately achieving convergence within 20 iterations. On the Vehicle dataset, the ASNMF-SRP algorithm shows a rapid decline in the objective function value during the initial iterations, but subsequently, the rate of decrease slows down, ultimately achieving convergence around 40 iterations.

## 5. Conclusions

This paper proposes an autoencoder-like sparse non-negative matrix factorization with structure relationship preservation (ASNMF-SRP). By integrating the principle of autoencoders, the algorithm enhances the optimization of the coefficient matrix through a linear “decoder-encoder” approach, which not only improves the factorization stability of the coefficient matrix but also further uncovers the latent representations of the original data. In the structural learning of the sample space, ASNMF-SRP effectively captures higher-order topological information between samples by constructing the optimal Laplacian matrix. In feature relationship learning, ASNMF-SRP employs the $l_{2,1}$-norm to define the reconstruction error of feature correlations between the basis matrix and the original data matrix, ensuring consistency in feature relationships between the low-dimensional space and the original high-dimensional space. Furthermore, a sparse constraint based on inner product representation is imposed on the coefficient matrix, further enhancing the clustering performance of ASNMF-SRP. Finally, comparative experiments between ASNMF-SRP and nine other advanced clustering algorithms demonstrate, as evidenced by the data in Table 4, Table 5, Table 6 and Table 7, that ASNMF-SRP achieves favorable clustering performance. In future research work, we will conduct in-depth exploration from the following three aspects: (1) To further optimize the construction process of high-order graph regularization, enhancing the model’s capability to represent complex structures; (2) to extend ASNMF-SRP into a deep NMF framework, enabling better exploration of latent features in data; and (3) to design a multi-view extension model for ASNMF-SRP, thereby broadening the applicability of the algorithm.

## Figures and Tables

**Table 1:** Introduction to five common sparsity methods.

No.	Method	Introduction to Sparsity Method
1	$l_{0}$-norm [34]	${\left\|V\right\|}_{0}$ denotes the number of non-zero elements in V.
2	$l_{1/2}$-norm [35]	${\left\|V\right\|}_{1/2}=\sum_{i=1}^{r}\sum_{j=1}^{n}{\left|v_{ij}\right|}^{1/2}$
3	$l_{1}$-norm [22]	${\left\|V\right\|}_{1}=\sum_{i=1}^{r}\sum_{j=1}^{n}\left|v_{ij}\right|$
4	$l_{2,1}$-norm [36]	${\left\|V\right\|}_{2,1}=\sum_{j=1}^{n}\sqrt{\sum_{i=1}^{r}v_{ij}^{2}}$
5	$l_{\log}$-(pseudo) norm [24]	${\left\|V\right\|}_{\log}=\sum_{i=1}^{r}\sum_{j=1}^{n}\log\left(1+\left|v_{ij}\right|\right)$

**Table 2:** Introduction of the 8 datasets.

NO.	Dataset	Samples (n)	Features (m)	Classes (r)	Data Type	Image Size
1	MSRA25	1799	256	12	Face dataset	16 × 16
2	Semeion	1593	256	10	Digit images	16 × 16
3	COIL20	1440	1024	20	Object images	32 × 32
4	COIL100	7200	1024	100	Object images	32 × 32
5	Krvs	3196	36	2	Network detection	—
6	Hitech	2301	2216	6	Technology news	—
7	PenDigits	3498	16	10	Handwritten digits	—
8	Vehicle	846	18	4	Vehicle contours	—

**Table 3:** Parameter values of ASNMF-SRP on different datasets.

No.	Dataset	Higher-Order Graph Regularization α	Feature Relationship Preservation β	Sparse Constraint λ
1	MSRA25	1000	1000	0.001
2	Semeion	1	100	0.0001
3	COIL20	1000	1000	0.001
4	COIL100	1000	1000	0.001
5	Krvs	100	10	0.0001
6	Hitech	1000	10	0.0001
7	PenDigits	100	100	0.001
8	Vehicle	1	1	0.001

**Table 4:** ACC of 10 algorithms on 8 datasets (mean ± standard deviation).

	Algorithm	NMF	ONMF	Hx-NMF	EMMF	GNMF	RMNMF	DRCC	FR-NMF	LS-NMF	ASNMF-SRP	I-P
Dataset												
MSRA25		0.50842	0.49375	0.52026	0.50592	0.53938	0.55550	0.28974	0.51659	0.54019	**0.57904**	4.24%
		±0.023	±0.026	±0.029	±0.018	±0.032	±0.034	±0.029	±0.022	±0.022	±0.045	--
Semeion		0.52508	0.56959	0.50807	0.51965	0.59209	0.27916	0.61601	0.53540	0.60251	**0.68063**	10.49%
		±0.042	±0.032	±0.042	±0.040	±0.039	±0.057	±0.037	±0.037	±0.036	±0.050	--
COIL20		0.66406	0.68531	0.65812	0.65142	0.76844	0.24385	0.79017	0.65028	0.77361	**0.84174**	6.53%
		±0.029	±0.028	±0.030	±0.019	±0.013	±0.094	±0.034	±0.026	±0.014	±0.012	--
COIL100		0.47026	0.49159	0.46877	0.47882	0.48738	0.41235	0.46099	0.47044	0.48306	**0.64173**	30.54%
		±0.014	±0.011	±0.012	±0.018	±0.014	±0.012	±0.014	±0.015	±0.011	±0.010	--
Krvs		0.51909	0.53742	0.52223	0.52137	0.53082	0.51810	0.55594	0.52552	0.53387	**0.56813**	2.19%
		±0.003	±0.012	±0.002	±0.003	±0.012	±0.007	±0.004	±0.023	±0.015	±0.003	--
Hitech		0.23385	0.23375	0.23403	0.23544	0.23105	**0.26273**	0.24087	0.22603	0.23268	0.25367	−3.45%
		±0.002	±0.005	±0.008	±0.004	±0.004	±0.001	±0.004	±0.007	±0.002	±0.002	--
PenDigits		0.66216	0.70729	0.68092	0.66630	0.67973	0.65183	0.73119	0.66791	0.68533	**0.80442**	10.02%
		±0.036	±0.048	±0.040	±0.038	±0.053	±0.037	±0.043	±0.034	±0.052	±0.032	--
Vehicle		0.38794	0.43777	0.40142	0.39096	0.44397	0.35916	0.41194	0.43570	0.44368	**0.45236**	1.89%
		±0.019	±0.002	±0.026	±0.021	±0.008	±0.003	±0.017	±0.023	±0.009	±0.002	--

**Table 5:** ARI of 10 algorithms on 8 datasets (mean ± standard deviation).

	Algorithm	NMF	ONMF	Hx-NMF	EMMF	GNMF	RMNMF	DRCC	FR-NMF	LS-NMF	ASNMF-SRP	I-P
Dataset												
MSRA25		0.34575	0.32933	0.35734	0.33482	0.40605	0.40058	0.12013	0.35562	0.40596	**0.44662**	9.99%
		±0.026	±0.027	±0.027	±0.020	±0.037	±0.034	±0.023	±0.019	±0.034	±0.052	--
Semeion		0.31198	0.35051	0.30655	0.31134	0.44280	0.11855	0.41651	0.31943	0.45921	**0.48809**	6.29%
		±0.033	±0.024	±0.031	±0.030	±0.032	±0.047	±0.026	±0.029	±0.030	±0.034	--
COIL20		0.57989	0.62582	0.57627	0.57069	0.74160	0.16450	0.73372	0.56682	0.74234	**0.80244**	8.10%
		±0.026	±0.023	±0.034	±0.026	±0.018	±0.093	±0.036	±0.024	±0.016	±0.007	--
COIL100		0.39584	0.44157	0.39549	0.40697	0.42371	0.30329	0.39167	0.39709	0.42067	**0.53573**	21.32%
		±0.016	±0.014	±0.017	±0.019	±0.010	±0.015	±0.016	±0.012	±0.011	±0.016	--
Krvs		0.00107	0.00579	0.00158	0.00147	0.00393	−0.00038	0.01201	0.00410	0.00505	**0.01814**	51.04%
		±0.000	±0.004	±0.000	±0.000	±0.003	±0.001	±0.002	±0.006	±0.005	±0.002	--
Hitech		−0.00095	0.00092	0.00021	0.00034	−0.00041	0.00016	0.00252	0.00066	−0.00049	**0.00689**	173.41%
		±0.001	±0.001	±0.001	±0.001	±0.001	±0.001	±0.002	±0.001	±0.001	±0.000	--
PenDigits		0.52361	0.55428	0.52352	0.52329	0.54137	0.50238	0.57474	0.52889	0.55809	**0.68487**	19.16%
		±0.029	±0.040	±0.037	±0.022	±0.045	±0.049	±0.037	±0.024	±0.039	±0.035	--
Vehicle		0.08169	0.12462	0.09207	0.08322	0.13103	0.06364	0.09691	0.11321	**0.13221**	0.12014	−9.13%
		±0.013	±0.003	±0.018	±0.018	±0.009	±0.004	±0.014	±0.012	±0.007	±0.001	--

**Table 6:** NMI of 10 algorithms on 8 datasets (mean ± standard deviation).

	Algorithm	NMF	ONMF	Hx-NMF	EMMF	GNMF	RMNMF	DRCC	FR-NMF	LS-NMF	ASNMF-SRP	I-P
Dataset												
MSRA25		0.56935	0.56296	0.57773	0.55467	0.65111	0.60295	0.23745	0.57715	0.64613	**0.71512**	9.83%
		±0.021	±0.028	±0.023	±0.021	±0.031	±0.029	±0.033	±0.017	±0.031	±0.026	--
Semeion		0.44162	0.48847	0.44312	0.44938	0.60790	0.20171	0.54014	0.44938	0.61489	**0.63282**	2.92%
		±0.025	±0.020	±0.026	±0.023	±0.020	±0.074	±0.018	±0.022	±0.019	±0.021	--
COIL20		0.76112	0.79591	0.76067	0.75423	0.88538	0.31375	0.89131	0.75546	0.88500	**0.91529**	2.69%
		±0.015	±0.010	±0.018	±0.017	±0.012	±0.131	±0.011	±0.015	±0.012	±0.006	--
COIL100		0.75258	0.76835	0.75400	0.75646	0.77226	0.70061	0.74641	0.73117	0.76948	**0.83835**	8.56%
		±0.005	±0.005	±0.006	±0.006	±0.004	±0.009	±0.006	±0.005	±0.004	±0.003	
Krvs		0.00060	0.00397	0.00091	0.00094	0.00265	0.00203	0.00818	0.00592	0.00352	**0.01250**	52.81%
		±0.000	±0.003	±0.000	±0.000	±0.002	±0.003	±0.001	±0.006	±0.003	±0.001	--
Hitech		0.00799	0.00989	0.00854	0.01049	0.00865	0.00558	0.01203	0.00786	0.00799	**0.01935**	60.85%
		±0.001	±0.002	±0.002	±0.001	±0.002	±0.001	±0.002	±0.002	±0.002	±0.000	--
PenDigits		0.68251	0.69332	0.66576	0.67684	0.69751	0.64294	0.69615	0.68480	0.70953	**0.80120**	12.92%
		±0.022	±0.019	±0.026	±0.018	±0.027	±0.043	±0.016	±0.021	±0.018	±0.021	--
Vehicle		0.11678	0.19000	0.13648	0.12714	0.19062	0.08408	0.14858	0.16404	**0.19181**	0.18544	−3.32%
		±0.014	±0.006	±0.019	±0.018	±0.015	±0.005	±0.019	±0.018	±0.013	±0.000	--

**Table 7:** PUR of 10 algorithms on 8 datasets (mean ± standard deviation).

	Algorithm	NMF	ONMF	Hx-NMF	EMMF	GNMF	RMNMF	DRCC	FR-NMF	LS-NMF	ASNMF-SRP	I-P
Dataset												
MSRA25		0.52985	0.52176	0.53755	0.52287	0.56587	0.57918	0.30698	0.53849	0.56651	**0.61479**	6.15%
		±0.021	±0.023	±0.025	±0.020	±0.026	±0.027	±0.030	±0.017	±0.025	±0.035	--
Semeion		0.53763	0.58804	0.53431	0.53807	0.63726	0.28738	0.63625	0.54862	0.64369	**0.69739**	8.34%
		±0.036	±0.025	±0.030	±0.031	±0.024	±0.061	±0.027	±0.028	±0.026	±0.031	--
COIL20		0.69135	0.71340	0.68628	0.67997	0.80715	0.24903	0.82802	0.67753	0.80892	**0.86455**	4.41%
		±0.024	±0.023	±0.021	±0.021	±0.017	±0.095	±0.017	±0.023	±0.018	±0.011	--
COIL100		0.52663	0.54705	0.52816	0.53379	0.54654	0.47951	0.51849	0.51637	0.54247	**0.69599**	27.23%
		±0.013	±0.010	±0.011	±0.013	±0.012	±0.012	±0.012	±0.012	±0.009	±0.006	--
Krvs		0.52245	0.53742	0.52289	0.52261	0.53137	0.52237	0.55594	0.53360	0.53387	**0.56813**	2.19%
		±0.000	±0.012	±0.001	±0.000	±0.011	±0.000	±0.004	±0.016	±0.015	±0.003	--
Hitech		0.26693	0.27034	0.26758	0.27017	0.26877	0.26380	0.27099	0.26788	0.26606	**0.28525**	5.26%
		±0.003	±0.003	±0.003	±0.003	±0.003	±0.001	±0.004	±0.005	±0.003	±0.001	--
PenDigits		0.69262	0.72340	0.69447	0.69118	0.70675	0.67226	0.73872	0.69626	0.71256	**0.81095**	9.78%
		±0.026	±0.031	±0.031	±0.026	±0.035	±0.035	±0.029	±0.024	±0.035	±0.023	--
Vehicle		0.39285	0.43777	0.40573	0.39681	0.44397	0.37145	0.41832	0.43853	0.44368	**0.45236**	1.89%
		±0.015	±0.002	±0.020	±0.018	±0.008	±0.005	±0.020	±0.022	±0.009	±0.002	--

**Table 8:** Clustering results of ASNMF-SRP and ASNMF-SRP-1 (mean ± standard deviation).

Dataset	ACC		ARI		NMI		PUR	
	ASNMF-SRP-1	ASNMF-SRP	ASNMF-SRP-1	ASNMF-SRP	ASNMF-SRP-1	ASNMF-SRP	ASNMF-SRP-1	ASNMF-SRP
MSRA25	0.49680	**0.57904**	0.33356	**0.44662**	0.55612	**0.71512**	0.51656	**0.61479**
	±0.021	±0.045	±0.016	±0.052	±0.019	±0.026	±0.019	±0.035
Semeion	0.64724	**0.68063**	0.48198	**0.48809**	**0.64292**	0.63282	0.68908	**0.69739**
	±0.010	±0.050	±0.007	±0.034	±0.010	±0.021	±0.011	±0.031
COIL20	0.80552	**0.84174**	0.76313	**0.80244**	0.88651	**0.91529**	0.83587	**0.86455**
	±0.015	±0.012	±0.017	±0.007	±0.008	±0.006	±0.013	±0.011
COIL100	0.48007	**0.64173**	0.40311	**0.53573**	0.73255	**0.83835**	0.52556	**0.69599**
	±0.009	±0.010	±0.011	±0.016	±0.005	±0.003	±0.007	±0.006
Krvs	0.52839	**0.56813**	0.00393	**0.01814**	0.00396	**0.01250**	0.53339	**0.56813**
	±0.017	±0.003	±0.004	±0.002	±0.004	±0.001	±0.012	±0.003
Hitech	0.23301	**0.25367**	0.00014	**0.00689**	0.00893	**0.01935**	0.26847	**0.28525**
	±0.005	±0.002	±0.001	±0.000	±0.001	±0.000	±0.003	±0.001
PenDigits	0.66533	**0.80442**	0.53130	**0.68487**	0.68684	**0.80120**	0.69495	**0.81095**
	±0.040	±0.032	±0.030	±0.035	±0.020	±0.021	±0.026	±0.023
Vehicle	0.44746	**0.45236**	**0.13445**	0.12014	**0.20032**	0.18544	0.44888	**0.45236**
	±0.004	±0.002	±0.003	±0.001	±0.008	±0.000	±0.006	±0.002

**Table 9:** Clustering results of ASNMF-SRP and ASNMF-SRP-2 (mean ± standard deviation).

Dataset	ACC		ARI		NMI		PUR	
	ASNMF-SRP-2	ASNMF-SRP	ASNMF-SRP-2	ASNMF-SRP	ASNMF-SRP-2	ASNMF-SRP	ASNMF-SRP-2	ASNMF-SRP
MSRA25	0.57518	**0.57904**	0.44437	**0.44662**	**0.72012**	0.71512	0.61354	**0.61479**
	±0.032	±0.045	±0.038	±0.052	±0.019	±0.026	±0.025	±0.035
Semeion	0.67803	**0.68063**	0.48380	**0.48809**	0.62883	**0.63282**	0.69567	**0.69739**
	±0.044	±0.050	±0.029	±0.034	±0.018	±0.021	±0.025	±0.031
COIL20	0.83865	**0.84174**	0.80155	**0.80244**	0.91285	**0.91529**	0.86194	**0.86455**
	±0.013	±0.012	±0.008	±0.007	±0.004	±0.006	±0.013	±0.011
COIL100	0.63818	**0.64173**	**0.54346**	0.53573	0.83103	**0.83835**	0.69085	**0.69599**
	±0.007	±0.010	±0.014	±0.016	±0.003	±0.003	±0.004	±0.006
Krvs	**0.56884**	0.56813	**0.01850**	0.01814	**0.01274**	0.01250	**0.56884**	0.56813
	±0.000	±0.003	±0.000	±0.002	±0.000	±0.001	±0.000	±0.003
Hitech	0.24744	**0.25367**	0.00424	**0.00689**	0.01893	**0.01935**	0.27912	**0.28525**
	±0.001	±0.002	±0.001	±0.000	±0.000	±0.000	±0.001	±0.001
PenDigits	0.79936	**0.80442**	0.67517	**0.68487**	0.79476	**0.80120**	0.80499	**0.81095**
	±0.029	±0.032	±0.028	±0.035	±0.017	±0.021	±0.022	±0.023
Vehicle	0.45219	**0.45236**	**0.12018**	0.12014	0.18540	**0.18544**	0.45219	**0.45236**
	±0.001	±0.002	±0.001	±0.001	±0.001	±0.000	±0.001	±0.002

## Data Availability

Publicly available datasets were analyzed in this study.

## References

[1] Kim S., Shin W., Kim H.W. (2024). Predicting online customer purchase: The integration of customer characteristics and browsing patterns. Decis. Support Syst..

[2] Zeng Y., Chen J., Pan Z., Yu W., Yang Y. (2025). Integrating single-cell multi-omics data through self-supervised clustering. Appl. Soft Comput..

[3] Mardani K., Maghooli K., Farokhi F. (2025). Segmentation of coronary arteries from X-ray angiographic images using density based spatial clustering of applications with noise (DBSCAN). Biomed. Signal Process. Control.

[4] Wold S., Esbensen K., Geladi P. (1987). Principal component analysis. Chemom. Intell. Lab. Syst..

[5] Zhang Q., Wang Y., Levine M.D., Yuan X., Wang L. (2015). Multisensor video fusion based on higher order singular value decomposition. Inf. Fusion.

[6] Lee D.D., Seung H.S. (1999). Learning the parts of objects by non-negative matrix factorization. Nature.

[7] Hoyer P.O. (2004). Non-negative matrix factorization with sparseness constraints. J. Mach. Learn. Res..

[8] Kong D., Ding C., Huang H. Robust Nonnegative Matrix Factorization Using L21-norm. Proceedings of the 20th ACM International Conference on Information and Knowledge Management.

[9] Ding C., Li T., Peng W. Orthogonal nonnegative matrix t-factorizations for clustering. Proceedings of the 12th ACM SIGKDD International Conference on Knowledge Discovery and Data Mining.

[10] Belkin M., Niyogi P., Sindhwani V. (2006). Manifold Regularization: A Geometric Framework for Learning from Labeled and Unlabeled Examples. J. Mach. Learn. Res..

[11] Cai D., He X., Han J., Huang T.S. (2010). Graph Regularized Nonnegative Matrix Factorization for Data Representation. IEEE Trans. Pattern Anal. Mach. Intell..

[12] Wu B., Wang E., Zhu Z., Chen W., Xiao P. (2018). Manifold NMF with L2,1 norm for clustering. Neurocomputing.

[13] Li X., Cui G., Dong Y. (2017). Graph Regularized Non-Negative Low-Rank Matrix Factorization for Image Clustering. IEEE Trans. Cybern..

[14] Liu Z., Zhu F., Xiong H., Chen X., Pelusi D., Vasilakos A.V. (2025). Graph regularized discriminative nonnegative matrix factorization. Eng. Appl. Artif. Intell..

[15] Huang S., Xu Z., Zhao K., Ren Y. (2020). Regularized nonnegative matrix factorization with adaptive local structure learning. Neurocomputing.

[16] Ren X., Yang Y. (2025). Semi-supervised symmetric non-negative matrix factorization with graph quality improvement and constraints. Appl. Intell..

[17] Mohammadi M., Berahmand K., Azizi S., Sheikhpour R., Khosravi H. (2025). Semi-Supervised Adaptive Symmetric Nonnegative Matrix Factorization for Multi-View Clustering. IEEE Trans. Netw. Sci. Eng..

[18] Defferrard M., Bresson X., Vandergheynst P. (2016). Convolutional neural networks on graphs with fast localized spectral filtering. Adv. Neural Inf. Process. Syst..

[19] Wang D., Ren F., Zhuang Y., Liang C. (2025). Robust high-order graph learning for incomplete multi-view clustering. Expert Syst. Appl..

[20] Zhan S., Jiang H., Shen D. (2025). Co-regularized optimal high-order graph embedding for multi-view clustering. Pattern Recogn..

[21] Wang Y., Yao H., Zhao S. (2016). Auto-encoder based dimensionality reduction. Neurocomputing.

[22] Chen Y., Qu G., Zhao J. (2024). Orthogonal graph regularized non-negative matrix factorization under sparse constraints for clustering. Expert Syst. Appl..

[23] Meng Y., Shang R., Jiao L., Zhang W., Yang S. (2018). Dual-graph regularized non-negative matrix factorization with sparse and orthogonal constraints. Eng. Appl. Artif. Intell..

[24] Peng C., Zhang Y., Chen Y., Kang Z., Chen C., Cheng Q. (2022). Log-based sparse nonnegative matrix factorization for data representation. Knowl.-Based Syst..

[25] Xiong W., Ma Y., Zhang C., Liu S. (2025). Dual graph-regularized sparse robust adaptive non-negative matrix factorization. Expert Syst. Appl..

[26] Shang F., Jiao L.C., Wang F. (2012). Graph dual regularization non-negative matrix factorization for co-clustering. Pattern Recognit..

[27] Gu Q., Zhou J. Co-clustering on manifolds. Proceedings of the 15th ACM SIGKDD International Conference on Knowledge Discovery and Data Mining.

[28] Hedjam R., Abdesselam A., Melgani F. (2021). NMF with feature relationship preservation penalty term for clustering problems. Pattern Recognit..

[29] Salahian N., Tab F.A., Seyedi S.A., Chavoshinejad J. (2023). Deep Autoencoder-like NMF with Contrastive Regularization and Feature Relationship Preservation. Expert Syst. Appl..

[30] Lee D.D., Seung H.S. Algorithms for non-negative matrix factorization. Proceedings of the 14th International Conference on Neural Information Processing Systems.

[31] Sun B.J., Shen H., Gao J., Ouyang W., Cheng X. A non-negative symmetric encoder-decoder approach for community detection. Proceedings of the 2017 ACM Conference on Information and Knowledge Management.

[32] Li T., Zhang R., Yao Y., Liu Y., Ma J., Tang J. (2024). Graph regularized autoencoding-inspired non-negative matrix factorization for link prediction in complex networks using clustering information and biased random walk. J. Supercomput..

[33] Zhang H., Kou G., Peng Y., Zhang B. (2024). Role-aware random walk for network embedding. Inf. Sci..

[34] Hosein M., Massoud B.Z., Christian J. (2009). A Fast Approach for Overcomplete Sparse Decomposition Based on Smoothed l~(0) Norm. IEEE Trans. Signal Process..

[35] Lu X., Wu H., Yuan Y., Yan P., Li X. (2013). Manifold Regularized Sparse NMF for Hyperspectral Unmixing. IEEE Trans. Geosci. Remote Sens..

[36] Meng Y., Shang R., Jiao L., Zhang W., Yuan Y., Yang S. (2018). Feature selection based dual-graph sparse non-negative matrix factorization for local discriminative clustering. Neurocomputing.

[37] Wang J., Wang L., Nie F., Li X. (2021). A novel formulation of trace ratio linear discriminant analysis. IEEE Trans. Neural Netw. Learn. Syst..

[38] Wang Q., He X., Jiang X., Li X. (2022). Robust Bi-stochastic Graph Regularized Matrix Factorization for Data Clustering. IEEE Trans. Pattern Anal. Mach. Intell..

[39] Chen M., Li X. (2023). Entropy Minimizing Matrix Factorization. IEEE Trans. Neural Netw. Learn. Syst..

[40] Huang J., Nie F., Huang H., Ding C.H.Q. (2013). Robust Manifold Nonnegative Matrix Factorization. ACM Trans. Knowl. Discov. Data.

